# Dorsal raphe serotonin neurotransmission is required for the expression of nursing behavior and for pup survival

**DOI:** 10.1038/s41598-021-84368-6

**Published:** 2021-03-16

**Authors:** Aude Muzerelle, Mariano Soiza-Reilly, Cornelia Hainer, Pierre-Louis Ruet, Klaus-Peter Lesch, Michael Bader, Natalia Alenina, Sophie Scotto-Lomassese, Patricia Gaspar

**Affiliations:** 1 INSERM, Institut du Fer À Moulin, Sorbonne Université UMR-S 1270, Paris, France; 2 Instituto de Fisiología, Biología Molecular y Neurociencias (IFIBYNE), CONICET, Universidad de Buenos Aires, Buenos Aires, Argentina; 3 Max-Delbrück Center for Molecular Medecine (MDC), Berlin-Buch, Germany; 4 Division of Molecular Psychiatry, Laboratory of Translational Neuroscience, Center of Mental Health, Department of Psychiatry, University of Würzburg, Würzburg, Germany; 5 Laboratory of Psychiatric Neurobiology, Institute of Molecular Medicine, I.M. Sechenov First Moscow State Medical University, Moscow, Russia; 6 Department of Neuropsychology and Psychiatry, School for Mental Health and Neuroscience (MHeNS), Maastricht University, Maastricht, The Netherlands; 7 German Center for Cardiovascular Research (DZHK), Partner Site, Berlin, Germany; 8 Charite-University Medicine, Berlin, Germany; 9 Institute for Biology, University of Lübeck, Lübeck, Germany; 10 Institute of Translational Biomedicine, St. Petersburg State University, St. Petersburg, Russia; 11 Institute of Cytology, Russian Academy of Science, St. Petersburg, Russia; 12 INSERM U1127, Paris Brain Institute, 75013 Paris, France

**Keywords:** Neural circuits, Social neuroscience

## Abstract

Proper maternal care is an essential factor of reproductive success in mammals, involving a repertoire of behaviors oriented toward the feeding and care of the offspring. Among the neurotransmitters involved in the initiation of these behaviors, serotonin (5-HT) seems to play an important role. Here we compared pup-oriented maternal behaviors in mice with constitutive 5-HT depletion, the tryptophan hydroxylase 2-knock-out (Tph2-KO) and the Pet1-KO mice. We report that the only common pup-oriented defect in these 2 hyposerotoninergic models is a defective nursing in parturient mice and altered nursing-like (crouching) behavior in virgin mice, while pup retrieval defects are only present in Tph2-KO. Despite a normal mammary gland development and milk production, the defect in appropriate nursing is responsible for severe growth retardation and early lethality of pups born to hyposerotonergic dams. This nursing defect is due to acute rather constitutive 5-HT depletion, as it is reproduced by adult knockdown of Tph2 in the dorsal raphe nucleus in mothers with a prior normal maternal experience. We conclude that 5-HT innervation from the dorsal raphe is required for both the initiation and maintenance of a normal nursing behavior. Our findings may be related to observations of reduced maternal/infant interactions in human depression.

## Introduction

In mammals, maternal care has an essential role to ensure the survival and well-being of the offspring. Successful maternal care requires the expression of a large repertoire of offspring-directed behaviors to feed and protect the young before they become autonomous^1–3^. The initiation and maintenance of such a behavioral repertoire is driven by a complex neuroendocrine cascade triggered by pregnancy and is reinforced by interactions with the young. These hormonal and sensory signals are relayed to the neural circuits controlling different aspects of maternal behavior, and involve different neuromodulators^2,4^. In several pathological conditions, such as in postpartum depression in humans, these mechanisms of brain plasticity fail, resulting in inappropriate maternal behavior, posing serious risks to the offspring^5^. This raises the question of a possible role of the neurotransmitters implicated in mood control such as serotonin (5-HT) and the effects of 5-HT related treatments^6^.

5-HT neurotransmission is involved in the regulation of a wide range of physiological and psychological processes, many of which are relevant to maternal behavior^2,6,7^. The brainstem dorsal raphe nucleus (DRN) provides a dense 5-HT innervations to all the brain areas that have been involved in the neuroendocrine and motivational aspects of maternal behavior, such as the hypothalamus, preoptic area, or nucleus accumbens^2,4,8,9^. Early studies involving lesions of the raphe nuclei in rats emphasized a main role of 5-HT in prolactin release, suggesting a predominantly neuroendocrine effect^10^, but more recently, several genetic mouse models of 5-HT depletion showed alterations in several other aspects of pup-oriented behaviors, in particular defects in nesting, pup retrieval, huddling, and nursing that could collectively account for the increased lethality of pups born to 5-HT depleted dams^11–14^. However, several questions remain regarding the role of 5-HT neurotransmission in these behaviors. In the first place, the phenotypes reported differed substantially among studies, which could either be due to effects of the genetic background^15^ or to the extent and specificity of 5-HT depletion^13,16^. For instance, in a mouse model targeting *Pet1* (Pet1-KO), the key transcription factor of 5-HT neuron specification, there is a failure in DRN neuron development, with a concomitant reduction of 5-HT by 75%, but also of its co-neurotransmitters, glutamate and various neuropeptides^17–19^. Conversely, in mouse models targeting central 5-HT synthesis (Tph2-KO) or its vesicular storage (conditional KO of *Slc18a2* in serotonergic neurons), raphe neurons develop normally and only 5-HT neurotransmission is reduced^11,20,21^. Another general issue in these genetic models is that 5-HT depletion is constitutive, affecting all central 5-HT systems; thus it remains unclear whether the observed defects are due to the lack of 5-HT per se or to altered brain development^16,22^. Moreover, given the heterogeneity of central 5-HT neurons and their different pattern of connectivity^17,23,24^, the specific neural 5-HT circuits responsible for these phenotypes are not known.

To clarify these issues, we compared pup-oriented maternal behaviors in three different genetic models of reduced 5-HT transmission in mice: two models with constitutive 5-HT depletion, where the extent and mechanism of central 5-HT depletion differ (the Pet1-KO and Tph2-KO), and a model of conditional 5-HT depletion in DRN neurons, using the Tph2-floxed mouse line (Tph2^flox/flox^)^20^ combined with stereotaxic viral delivery of Cre-recombinase in adulthood. Our study confirms major defects in the survival of progeny from constitutive hyposerotonergic models and extends previous findings by providing a comprehensive and more detailed description of maternal behaviors in both parturient (primiparous and secondiparous) and virgin females in mice of the same C57BL/6 background. Further, we demonstrate that selective depletion of DRN 5-HT is sufficient to reproduce the phenotype of the Pet1-KO mice. Overall, this study showed that defective nursing behavior is shared by all female mice with a central 5-HT depletion. Importantly, alterations of pup-oriented behaviors appeared to be correlated to the severity of 5-HT reduction, rather than to differences in the mechanism of 5-HT depletion or to developmental mechanisms.

## Materials and methods

### Animals

Animal experiments and handling followed in France the French Agriculture and Forestry Ministry guidelines for Handling Animals (decree 87849) and in Germany the National Institutes of Health Guide for the Care and the Use of Laboratory Animals. Protocols were approved by the Charles Darwin ethical committee (agreements 09047 and 09162, Paris) and the ethical committee of the local government (LAGeSo, Berlin). All experiments were conducted in accordance with the standard ethical guidelines of the European Community and complied with the ARRIVE guidelines.

All animals were bred under standard laboratory conditions (22 ± 1 °C, 60% relative humidity, 12 h dark/light cycle, food and water ad libitum). Females without pups were group-housed (3–5 per cage) and were at least 12 weeks of age on the first mating^15^. All pregnant females were isolated one week before delivery and were given a nestlet (5 cm^2^ of cellulose, SAFE, France) to assemble a nest. Virgin females tested for pup interaction were similarly isolated 3 days before testing.

The Pet1-KO mouse strain is a gift from Evan Deneris (Case Western Reserve University, Cleveland, OH) originally produced on a mixed 129 Sv and C57BL/6 background^12,18^. It has since been backcrossed for over 10 generations to the C57BL/6 background^19^. Pet1-KO males were crossed with heterozygous (Pet1^+/−^) females providing mutant and control female littermates. The choice of Pet1^+/−^ littermates as controls, was based on previous studies showing that there is no difference in the number 5-HT neurons and in 5-HT levels between wild-type (WT) and Pet1^+/−^^18,19^. Additionally, behavioral analyses showed they are indistinguishable^19,25^.

The generation of the TPH2-KO mouse line has been described^11^. These mice have, since the original description, been backcrossed to a pure C57BL/6 genetic background^26^. Here, Tph2^+/+^ were used as controls, because the Tph2^+/−^ have been noted to display behavioral changes^27^.

The conditional Tph2^flox/flox^ line, containing the Cre-exciseable loxP sequences that target exon 5, were generated on a mixed 129 Sv and C57BL/6 genetic background^20,28^, but later backcrossed to a pure C57BL/6 genetic background (Tph2^tm1.1Kpl^; K.P. Lesch, to whom correspondence regarding this mouse line should be addressed).

### AAV injections in the Dorsal Raphe Nucleus (DRN)

The replication-defective adeno-associated virus (AAV9.hSyn.HI.eGFPLCre.WPRE.SV40; 10^12^–10^13^ genome containing particles/ml; Penn Vector Core, USA) was used to obtain the expression of the Cre recombinase and the green fluorescent protein (GFP) in the DRN neurons.

Stereotaxic injections were performed as described^8^. The volume and titer of virus was optimized in pilot experiments for efficient recombination in the 5-HT neurons: 300 nl of the viral solution (1/100 in NaCl 0.9%) was injected per site in the DRN (+ 0.5 anterior to the lambda, ± 0.7 lateral, and -3.2 ventral from the brain surface with a 10° angle). Tph2^flox/flox^ females undergoing surgery (DRN^Tph2−CKO^) were allowed a recovery period of 3 weeks before mating. Wild-type C57BL/6 mice (local breeding and Janvier Labs, France) were used as controls (DRN^control^).

### Behavioral studies on postpartum females

Female mice were mated with WT C57BL/6 males. The day of the vaginal plug was considered as E0, and the delivery generally occurred early morning at E19.

Maternal behavior was evaluated the day of delivery (designated as postpartum day 0, PPD0) during the light phase, and pup parameters were followed up between PPD0 and PPD7. Care was taken to minimize dam’s stress: 1 h minimum after the end of parturition, the cage was carefully moved to a quiet room to perform observations. The pups were removed only when the dam had moved away from the nest and the litter was culled to 5 pups after the pup retrieval assay.

#### Observation in the home cage

Maternal behavior was assessed at PPD0 during 20 min observations divided into 4 sessions of 5 min each (from 10 am to 5 pm) for all mice, except for the Tph2^−/−^, for which only 3 sessions could be performed. Time spent outside the nest and time dedicated to pup-directed responses inside the nest (1/ nursing, i.e. when the mother is crouching over the pups with or without a clear arched-back posture, and 2/ pup licking) were recorded. Data were analyzed as the percentage of time during which animals engaged in each activity.

#### Pup retrieval assay

This was done at the end of the second observation session at PPD0. All the litter was removed from the home cage for 5 min. Then 5 pups were placed back into the home cage at a distance (> 15 cm) from the nest. The latency to retrieve each pup and the number of pups brought back to the nest was noted during 10 min.

In independent cohorts of mice, the same test was performed after a mild stress. Dams were first isolated 10 min in a new cage without bedding before being introduced back in their home cage with dispersed pups. Delay and number of pups brought back to the nest were measured as above.

#### Nesting behavior

The quality of the nests was scored using the Deacon rating^29^ as follows: 1 = nestlet untouched, 2 = nestlet partially torn, 3 = nestlet completely shredded but no nest, 4 = flat nest, 5 = complete covered nest with high walls. The first nest rating was done few hours after delivery in the undisturbed home cage (PPD0). After the last session of observations, all nest material was removed from the cage and a new square of cellulose was deposited close to the pups. A second nest scoring was performed the morning of the following day (PPD1).

#### Huddling score

Pup location (inside or outside the nest) was recorded at PPD0 as an indicator of the huddling of pups with the following scale: score 1 = all the pups inside the nest, score 0.5 = 2 or more pups outside the nest, and 0 = all the pups outside the nest.

#### Pup development and feeding

Pups were checked for remaining amniotic membranes, umbilical cord or placenta attached to the body at birth and were weighed at PPD0, PPD1 and PPD2, noting possible bite marks, and scoring the milk pouch as follows: score 0 = no milk, score 1 = some milk visible; score 2 = clear milk pouch; score 3 = full milk pouch.

Pup survival was followed until PPD7 when possible (e.g. at least 1 pup surviving).

### Behavioral studies in virgin females (alloparental behavior)

Virgin females were individually housed 3 days prior to the experiment. During a session, each female was exposed to three 1- to 4-day-old pups from a C57BL/6 donor female, placed at distance from the nest^15^. The latencies to retrieve all the pups and to crouch over at least 2 pups continuously for 1 min or more were recorded during 30 min. After the test, donor pups were returned to their mother. The procedure was repeated every day for up to 4 (for Tph2-KO) or 5 (for Pet1-KO) consecutive days. At the end of the whole procedure, females were distributed in 4 categories: (1) infanticidal (attack to pups which led to interrupt the test), (2) non-responding (i.e. showing no interaction with pups), (3) retrieving all pups to the nest, eventually followed by (4) crouching over pups.

### Brain tissue processing

Anesthetized (Pentobarbital 0.5 mg/g) mice were fixed by intracardiac perfusion of 4% paraformaldehyde in 0.1 M phosphate-buffered saline (PBS; pH 7.4). Brains were post-fixed overnight in the same fixative and cryoprotected 2 days in 30% sucrose containing sodium azide (0.01%; Sigma-Aldrich Co., MO, USA). Coronal sections were prepared (50 µm thick) on a cryo-microtome (Microm Microtech, France). Serial sections of the raphe nuclei were collected as series of 3.

Triple immunofluorescent labeling was performed as previously described^8^ and carried out using primary antibodies against GFP (1/1000 Aves Labs, USA; GFP-1020), 5-HT (1/5000, Sigma-Aldrich, USA; S5545) or TPH (1/1000 Millipore, USA; AB1541). The corresponding fluorescent secondary antisera used were raised in donkey (1/200, Jackson ImmunoResearch, UK). Sections were mounted in Mowiol (10%, Calbiochem, Germany)-Dabco (2.5%, Sigma-Aldrich Co., MO, USA).

### Mammary gland processing

The 24hs prepartum (PPD-1) females and 48hs postpartum (PPD2) dams were euthanized and the fourth inguinal mammary gland pair was dissected as described^30^. Tissue was then fixed 2 hs in Carnoy’s fixative (100% ethanol, chloroform, glacial acetic acid; 6:3:1) at room temperature, washed during 2 hs in 100% ethanol, and then stored in 70% ethanol. Samples were weighed and embedded in paraffin for histological examination.

### Image acquisition and quantification

Fluorescent images were acquired with the same settings on a Leica DM6000 fluorescence microscope using a 20X /0.70 N.A. objective. Stitching of multiple micrographs into a single mosaic image was done with MetaMorph version7.8 (Molecular Devices, USA).

All cases with AAV injections were analyzed to check the localization and extent of injection. TPH2 + cells were counted on 4 rostro-caudal levels encompassing the 3 DRN subdivisions (DRNd: dorsal; DRNv: ventral; DRNl: lateral) using the Cell Counter plugin of Fiji software. Numbers refer to the total cell counts from 4 sections.

### Statistical analysis

Analyses were done using Prism version 9.0.0 for Windows (GraphPad software, La Jolla, CA, USA) and IBM SPSS 20.0. Distributions of two unmatched groups were compared with the two-tailed Mann–Whitney test (MW). Genotype comparisons for the pup retrieval assay and measures of pup growth parameters (weight and milk pouch) were performed using the repeated measures two- or three-way ANOVA with the Geisser-Greenhouse correction since sphericity was not assumed. For measures of mammary gland weight, comparisons between genotypes were done using two-way ANOVA. In all the ANOVAs, the normality of the datasets was verified: all distributions were normal except for the data on milk pouch which were log-transformed before doing statistical analysis. Multiple post-hoc comparisons were done by Tukey’s or Sidak’s or uncorrected Fisher’s LSD method. For contingency analyses, the two-sided exact Fisher test or the Pearson chi-square was used. Survival curves were analyzed by Log-rank Mantel-Cox method. Point-biserial correlations were applied to determine the relationship between pup survival, TPH2 + cell numbers and nursing times with the non-parametric two-tailed Kendall’s tau method. Other correlations were done with the two-tailed Spearman method. Each statistical analysis used is specified in the text or in the figure legends. All results are expressed as mean ± sem. Significance level was established at *p* ≤ 0.05.

## Results

### Post-partum maternal behavior is impaired in primiparous Pet1-KO and Tph2-KO mice

Previous studies indicated that pups born to Pet1-KO dams do not survive^12^ while litters of Tph2-KO show a 55% pup survival rate^11^. However, these studies were performed on mixed C57BL/6 and 129 Sv genetic backgrounds. We profited from the fact that both strains have since been backcrossed to pure C57BL/6 background^19,26^ to compare pup survival and post-partum maternal behaviors using more comparable protocols and conditions. Knock-out dams were mated to WT males to avoid possible interferences due to the effects of the pup’s genotype. Indeed hyposerotonergic pups are known to have growth defects and low survival rates, independently of the mother’s genotype^14,31^, whereas heterozygote Pet1^+/−^ and Tph2^+/−^ pups grow normally and show no difference in their survival rate compared to their WT littermates^18,31^.

**Figure 1:**
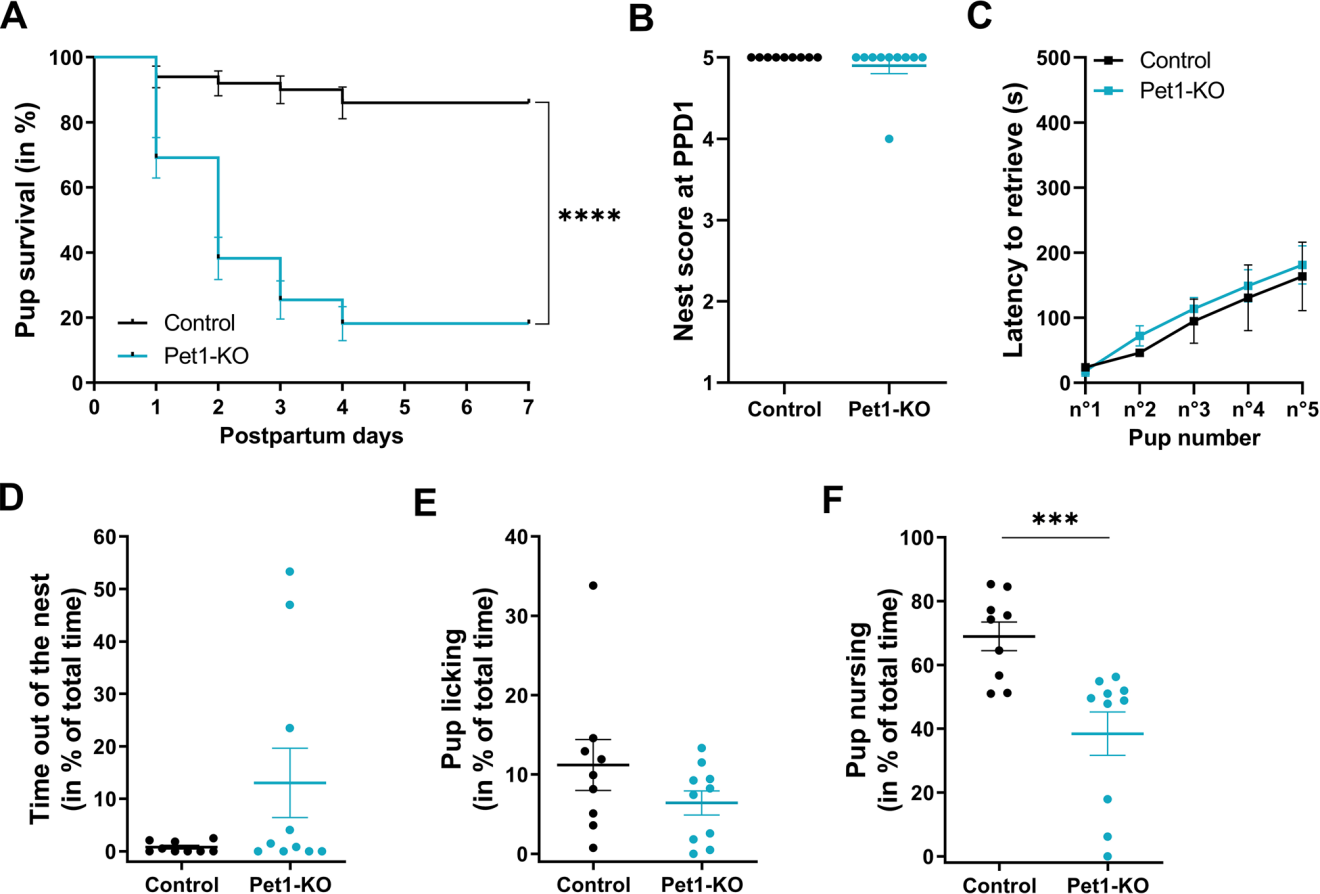
Reproductive success and nursing behavior are altered in primiparous Pet1-KO mothers. Nulliparous Pet1-KO and control (Pet1^+/−^) mice were mated to WT males. The number of litters analyzed was n = 11 for Pet1-KO dams and n = 10 for control dams. All litters were culled to 5 pups. (**A**) Survival of pups raised by Pet1-KO dams was strongly reduced; ****p < 0.0001 (mean ± SEM, Log-rank Mantel-Cox test). (**B**) Nest building was graded using the Deacon’s scale^29^ from score 1 = untouched to score 5 = complete nest with high walls. Nest building score at PPD1 was similar between Pet1-KO and control dams (mean ± SEM, MW U = 40.50, *p* > 0.99). (**C**) Latency to retrieve 5 pups into the nest in seconds over a 10 min period. No difference in the latency to retrieve pups was found (Pet1-KO: n = 10; controls: n = 9) (mean ± SEM, repeated-measures two-way ANOVA: genotype x pup number interaction F_4,68_ = 0.1862, *p* = 0.94; Genotype main effect F_1,17_ = 0.2253, *p* = 0.64). (**D–F**) Maternal behavior was scored at PPD0 during 20 min observations. Time spent outside the nest (**D**) and pup licking activity (**E**) did not differ between Pet1-KO and controls, but Pet1-KO dams spent significantly less time nursing (**F**); ***p < 0.001 (mean ± SEM, Mann–Whitney test).

Pet1-KO and control (Pet1^+/−^) littermate nulliparous females (n = 11 and n = 10 respectively) were used for this analysis. No differences in the rate and duration of pregnancy were noted between Pet1-KO and control dams. Initial litter sizes were comparable for both genotypes (n = 4–8 pups, culled into n = 5/per dam) which is similar to observations with standard C57BL/6 mice^32^. In addition, pups from both groups had clean skin, indicating normal placentophagia in Pet1-KO mice. However, notable differences in pup survival were noted by postnatal day 1 (PPD1) with an increased number of dead pups in litters born to Pet1-KO dams. At PPD7, pups born to control mice showed a 86 ± 4.9% survival rate which is in the normal range reported in other studies using primiparous C57BL/6 mice^33^. In contrast, only 18.2 ± 5.2% of pups born to Pet1-KO dams survived (Log-rank Mantel-Cox test: *p* < 0.0001; Fig. 1A). This strong lethality reflects entire offspring losses of 9/11 litters in Pet1-KO dams, against 1/10 litters in controls. Dead pups were generally found in the nest or close to it. Cannibalism was noted in 2/11 Pet1-KO mothers, but not in controls (Fisher’s exact test: *p* = 0.48). The nest quality did not differ, neither a few hours after delivery (mean score Pet1-KO: 4.8 ± 0.2 vs. controls: 5; MW U = 45, *p* > 0.99), nor the following day (Fig. 1B), indicating that maternal nesting behavior is preserved in Pet1-KO dams. Pup huddling was scored at PPD0 and no difference between genotypes was observed (Pet1-KO: 0.89 ± 0.11 vs. controls: 0.89 ± 0.07; MW U = 37, *p* > 0.99). In the pup retrieval assay, all tested dams (n = 10 Pet1-KO and n = 9 controls) were able to collect the 5 pups (MW U = 45, *p* > 0.99) with no difference in the latency to carry them to the nest (Fig. 1C). Because in a previous report, Pet1-KO mice showed a deficit in pup retrieval^12^, we evaluated a possible interaction with mild stress^15^. Using a new batch of mice (n = 6 Pet1-KO and n = 5 controls), pup retrieval was tested after a 10 min exposure to a novel cage without bedding. This mildly stressful manipulation reduced the percentage of retrieved pups, but the change was not significant (supplementary Fig. 1A) and the latency to bring back the pups inside the nest was unchanged (supplementary Fig. 1B). These experiments indicated that, on the C57BL/6 background, Pet1-KO dams show no deficits in pup retrieval. We then analyzed pup-related activities in undisturbed cages. Although a few Pet1-KO dams spent more time outside the nest, as a group this difference was not significant (MW U = 32, *p* = 0.29; Fig. 1D). Similarly, time devoted to pup licking was similar between both groups (MW U = 29, *p* = 0.21; Fig. 1E). In contrast, nursing behavior, defined as the time spent crouching over the pups with kyphosis posture, was significantly reduced in Pet1-KO dams (MW U = 6.5, *p* = 0.0007; Fig. 1F). Nursing in rodents comprises an alternation of two types of kyphosis, low and high-arched back postures. Both positions were observed in controls, but only the low-arched posture was noted in Pet1-KO dams. Altogether, these data indicated that Pet1-KO mothers on a pure C57BL/6 genetic background, show altered maintenance and survival of their litters, consistent to the original observations^12^. However, this was not related to general defects of maternal behaviors such as nesting, huddling or pup retrieval but was mainly associated with a deficit in nursing.

**Figure 2:**
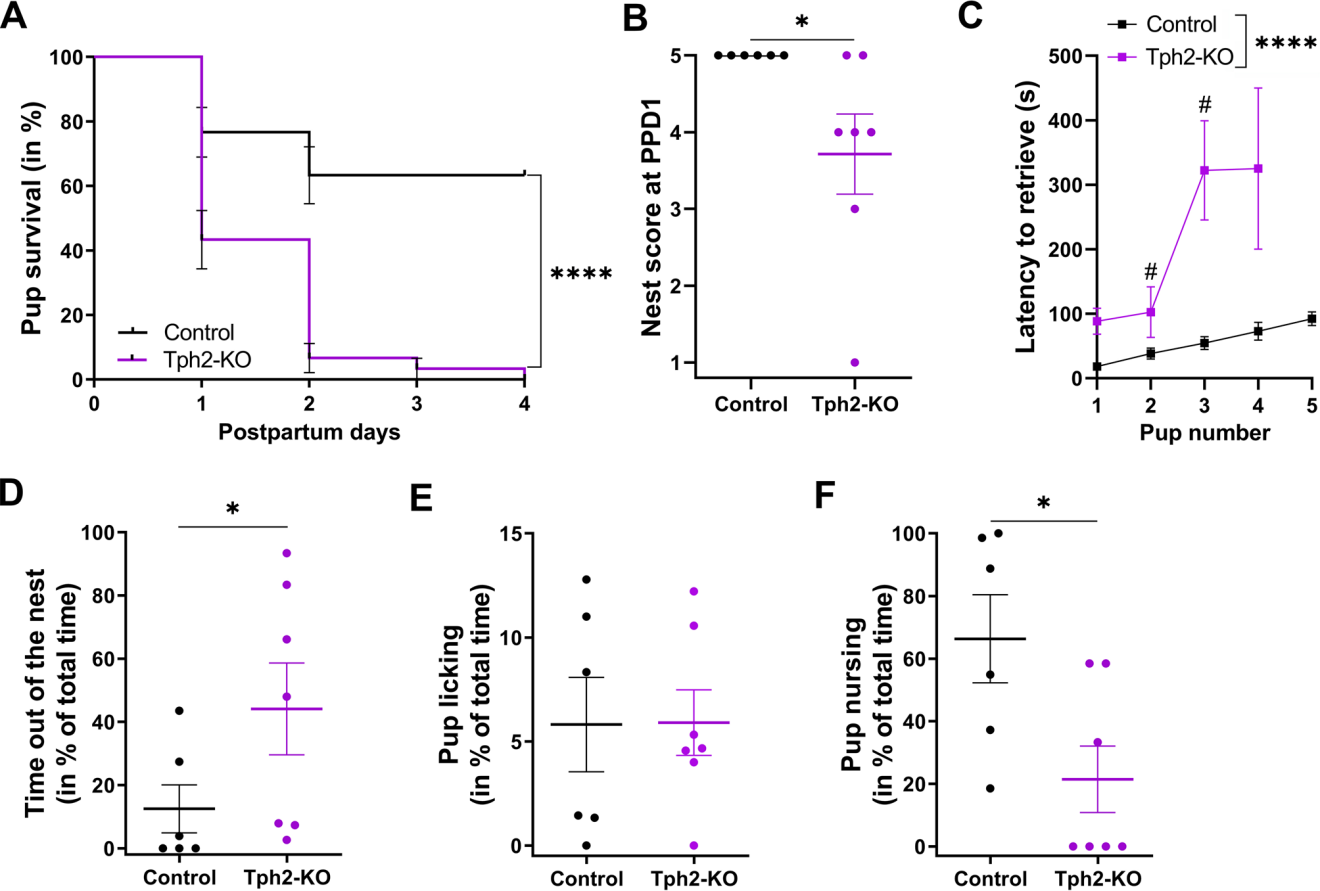
Reproductive success and maternal behaviors are altered in primiparous Tph2-KO mothers. Nulliparous Tph2-KO and control (Tph2^+/+^) mice were mated to WT males. The number of litters analyzed was n = 7 for Tph2-KO dams and n = 6 for control dams. All litters were culled to 5 pups. (**A**) Contrary to control dams, all the litters from Tph2-KO dams were lost by postpartum day 4; ****p < 0.0001 (mean ± SEM, Log-rank Mantel-Cox test). (**B**) Nest building scores at PPD1 were significantly lower in Tph2-KO dams than in controls, *p < 0.05 (mean ± SEM, Mann–Whitney test). (**C**) Latencies to retrieve pups were significantly reduced in Tph2-KO dams mean ± SEM, ****p < 0.0001 (repeated-measures two-way ANOVA followed by uncorrected Fisher’s LSD: ^#^*p* = 0.04). None of the Tph2-KO dams was able to retrieve the fifth pup, and 3/7 of Tph2-KO dams did not retrieve any pup and therefore could not be included in the quantification of the latency to retrieve. (**D**) Tph2-KO dams spent more time outside the nest compared to control dams; *p < 0.05 (mean ± SEM, Mann–Whitney test). (**E,F**) Tph2-KO and control dams dedicated the same time to pup licking (MW U = 20.5, *p* = 0.98), but Tph2-KO spent significantly less time nursing; *p < 0.05 (mean ± SEM, Mann–Whitney test).

We then looked at a model of more complete depletion of brain 5-HT, the Tph2-KO^11^. Tph2-KO and control (Tph2^+/+^) nulliparous females (n = 7 and n = 6 respectively) were used for this analysis. None of the litters born to Tph2-KO mothers survived beyond 4 days (Log-rank Mantel-Crox test: *p* < 0.0001; Fig. 2A). None of the mothers from both groups were found to be infanticidal, and no signs of cannibalism were noted. Nest building behavior of Tph2-KO was altered compared to control dams. After delivery, the nests of Tph2-KO mothers were less high than those of controls (mean score: Tph2-KO: 4.1 ± 0.3 vs. controls: 5; MW U = 6, *p* = 0.02). At PPD1, the nests of Tph2-KO dams were graded 3.7 ± 0.5, contrasting with the high-walled nests of control dams (mean score: 5; MW U = 6, *p* = 0.02; Fig. 2B). Pups from Tph2-KO mothers were generally scattered around the nest, also indicating a defect in huddling (scored in Tph2-KO: 0.36 ± 0.09 vs. controls: 0.92 ± 0.08; MW U = 2.5, *p* = 0.004). In the pup retrieval assay, 42.8% (3/7) of the Tph2-KO dams collected no pup over the 10 min period. The remaining females retrieved up to 4 pups but with an increased latency compared to controls (two-way ANOVA: Genotype x pup number interaction F_3,31_ = 7.83, *p* = 0.0005; Genotype main effect F_1,31_ = 57.77, *p* < 0.0001; Fig. 2C). Monitoring maternal activity in the home cage showed that Tph2-KO mothers spent significantly more time outside the nest than controls (MW U = 7, *p* = 0.05, Fig. 2D). Pup licking activity of Tph2-KO and control dams in the nest was similar (Fig. 2E), but the time dedicated to nursing was significantly reduced (MW U = 7, *p* = 0.05; Fig. 2F).

Overall, the evaluation of maternal behavior in these two mouse lines with constitutive reduction of 5-HT transmission, though of a different nature, showed some commonalities: both Tph2-KO and Pet1-KO dams showed poor survival of their litters and deficits in nursing behavior (supplementary Fig. 2) while other maternal behaviors were moderately altered in the Tph2-KO dams, but not in the Pet1-KO dams. In the absence of overt signs of maternal aggressive behaviors or of major changes of pup-directed behaviors such as nest building or pup licking, the defective crouching over pups thus appeared as the common feature explaining increased pup lethality.

### Previous maternal experience does not improve maternal behavior in Pet1-KO mice

Because pup-directed behavior and offspring survival improve with maternal experience in rodents^34^, we questioned whether this could also be the case of Pet1-KO mice. To test this, Pet1-KO (n = 8) and control (n = 10) dams that had been analyzed for primiparous maternal behavior were mated again to WT males. Although survival of these second litters was slightly improved (Pet1-KO: 29 ± 7.4% and controls: 91.8 ± 3.9%), this did not reach significance when compared to the first litter in either Pet1-KO (Log-rank Mantel-Cox test: *p* = 0.23) or controls (Log-rank Mantel-Cox test: *p* = 0.35) dams. On the other hand, the genotype difference was maintained (Log-rank Mantel-Cox test: *p* < 0.0001; supplementary Fig. 3A). Quality of the nest (supplementary Fig. 3B), mean huddling score and pup retrieval assay (supplementary Fig. 3C) were unchanged in the experienced Pet1-KO mothers. Similarly to primiparous dams, secondiparous Pet1-KO mothers showed a non-significant trend to spend more time outside the nest (MW U = 22.5, *p* = 0.13; supplementary Fig. 3D) and a significant reduction in nursing time (MW U = 15, *p* = 0.03; supplementary Fig. 3E), with a reduced occurrence of the high-arched back posture. Thus, maternal experience did not change maternal responsiveness in the Pet1-KO and confirmed that a defective nursing behavior was associated with a poor survival rate of the offspring.

**Figure 3:**
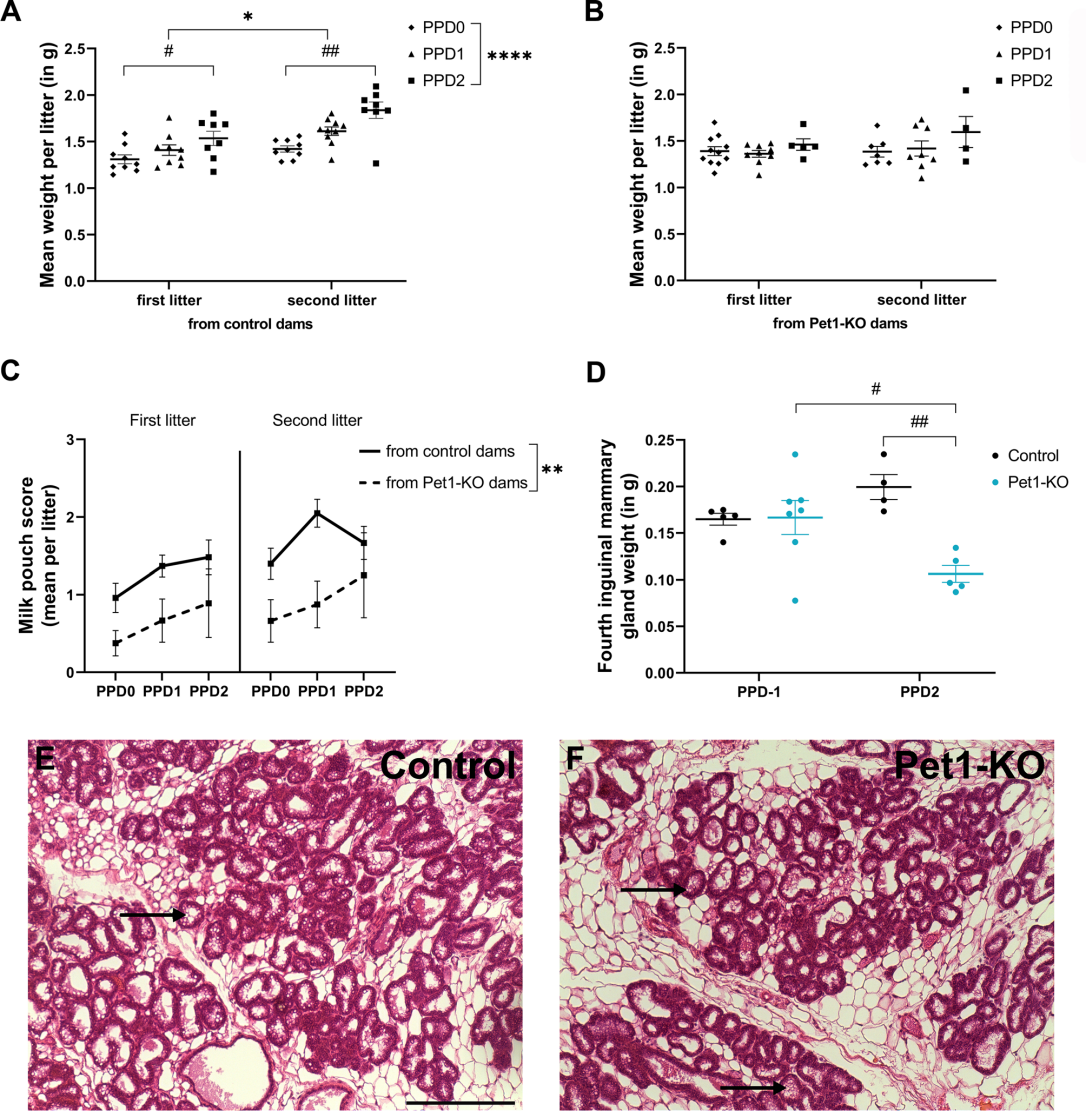
Defect in pup survival in the Pet1-KO mice is associated to reduced milk intake despite normal mammary gland development. (**A**) Pups of the first (n = 9) and the second litters (n = 10) raised by control (Pet1^+/−^) dams showed a significant weight gain during their first two postnatal days, with a significant effect of maternal experience; *p < 0.05, ****p < 0.0001 (mean per litter ± SEM, repeated-measures two-way ANOVA followed by Tukey’s comparisons: ^#^*p* = 0.011; ^##^*p* = 0.004). (**B**) Pups of the first (n = 10) and second litters (n = 8) raised by Pet1-KO dams did not show weight gain between PPD0 and PPD2, no matter the reproductive experience of the mothers; (mean per litter ± SEM; repeated-measures two-way ANOVA). (**C**) Milk pouch of pups born by Pet1-KO dams was consistently smaller than in pups of control dams. No matter the genotype of the dams, there was no significant change in the milk pouch size of their pups between PPD0 and PPD2, nor any effect of maternal experience, mean per litter ± SEM, **p < 0.01 (repeated-measures three-way ANOVA). (**D**) The weight of the mammary gland was measured at late gestation and after pup delivery. 24hs prepartum (PPD-1) the mammary gland weight was comparable between Pet1-KO and control mice. 48hs postpartum (PPD2), the mammary gland weight of Pet1-KO dams was significantly reduced compared to controls, as well as vs. the ones from PPD-1 Pet1-KO (mean ± SEM, two-way ANOVA followed by Tukey’s comparisons: ^#^*p* = 0.03; ^##^*p* = 0.003). (**E,F**) Hematoxylin/eosin-stained sections of mammary glands from PPD2 (**E**) controls and (**F**) Pet1-KO dams. Histological structure of the mammary tissue was comparable between mothers from both genotypes. Acini seemed smaller and less numerous in the Pet1-KO dams (**F**) compared to controls (**E**), but they contain small lipid droplets, that correspond to milk presence (black arrows). Scale bar = 200 µm.

### Nursing deficit of Pet1-KO dams results in poor development of their pups.

We evaluated pup development and feeding by measuring their daily growth and milk intake. At birth (PPD0), there was no difference in mean pup weight (supplementary Fig. 4A), indicating that the genotype and reproductive history of the mother had no effect on embryonic growth. Between PPD0 and PPD2, a significant weight increase was measured in litters of control dams, an effect that was also enhanced by maternal experience (two-way ANOVA: Maternal experience x Postnatal day interaction F_2,30_ = 2.996, *p* = 0.07; Postnatal day main effect F_1.390,20.85_ = 47.72, *p* < 0.0001; Maternal experience main effect F_1,17_ = 7.664, *p* = 0.01; Fig. 3A). In contrast, litters from Pet1-KO dams showed no significant growth between PPD0 and PPD2, whatever the reproductive experience (two-way ANOVA: Maternal experience x Postnatal day interaction F_2,21_ = 0.3404, *p* = 0.72; Postnatal day main effect F_1.581,16.61_ = 1.102, *p* = 0.34; Maternal experience main effect F_1,17_ = 0.3867, *p* = 0.54; Fig. 3B). Milk intake was evaluated by checking the presence of a milk pouch at PPD0, and by rating its size between PPD0 and PPD2 (supplementary Fig. 4B). In first litters, a milk pouch was present in 37% of the pups born to Pet1-KO dams versus 68% to control dams (Fisher’s exact test: *p* < 0.0001). In second litters, milk was noted in 78% of the pups born to Pet1-KO and 91% of the control dams (Fisher’s exact test: *p* = 0.003). Milk pouches were systematically smaller in pups raised by Pet1-KO dams between PPD0 and PPD2 compared to pups raised by control dams, irrespective of maternal experience (three-way ANOVA: Genotype x Postnatal day x Maternal experience interaction F_2,3_ = 0.4212, *p* = 0.69; Genotype main effect F_1,20_ = 12.29, *p* = 0.002; Postnatal main effect F_2,40_ = 2.68, *p* = 0.08; Maternal experience main effect F_0.7275,14.55_ = 0.5471, *p* = 0.42; Fig. 3C). Of note, pups born to Pet1-KO dams could be rescued by cross-fostering to control lactating dams, although this was not measured in a systematic manner.

**Figure 4:**
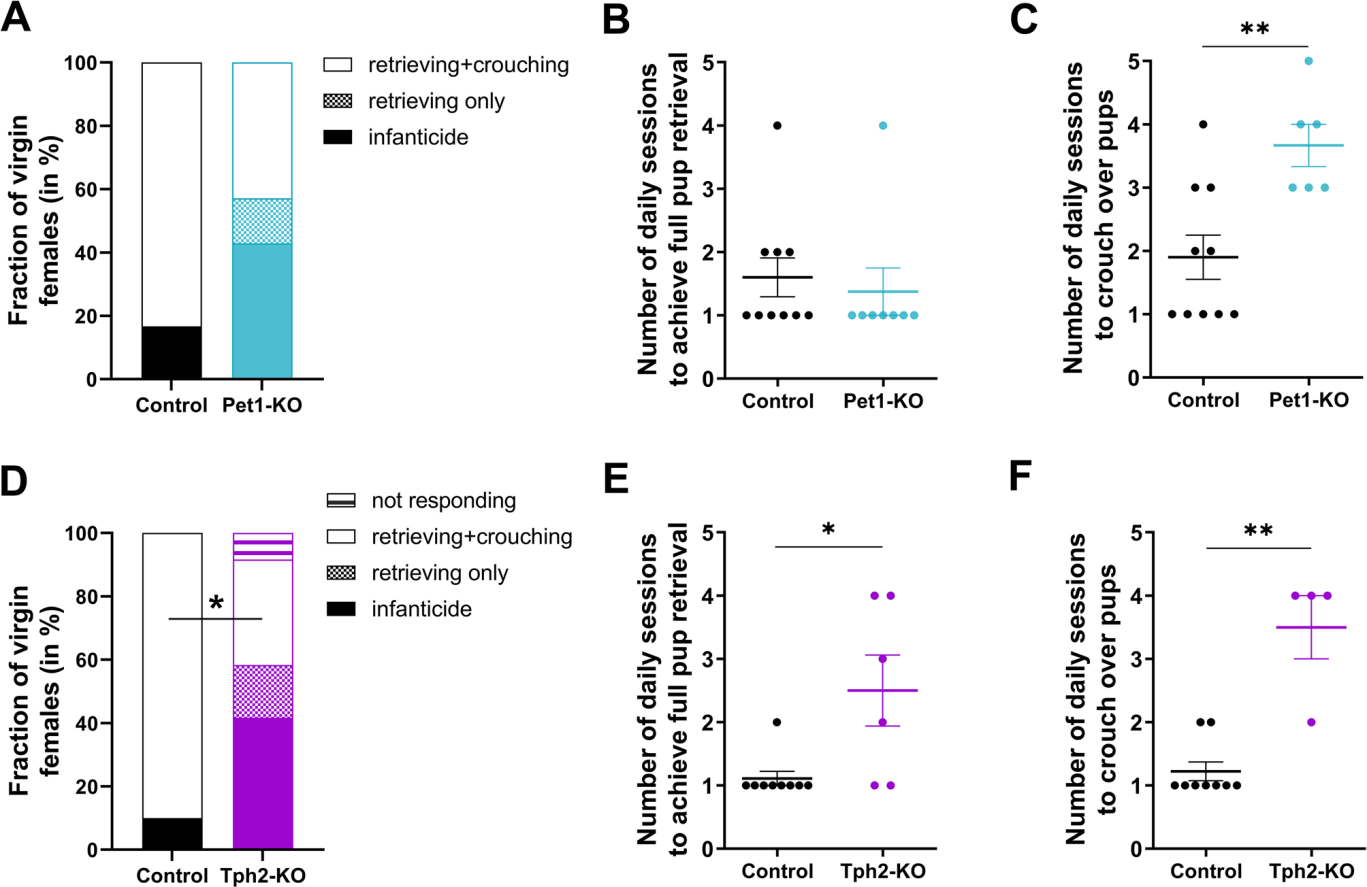
5-HT depletion is associated to a delayed initiation of crouching behavior onto pups in virgin mice. Virgin (**A–C**) Pet1-KO (n = 14) and (**D–F**) Tph2-KO (n = 12) females and their littermate controls (n = 12 Pet1^+/−^ and n = 10 Tph2^+/+^) were individually exposed in their home cages to three 1- to 4-day-old C57BL/6 pups. Training session lasted for 30 min and were repeated daily up to 4 or 5 days. (**A,D**) Histograms showing for each genotype, the distribution (in %) of the females at the end of the procedure in the following categories: infanticidal, not responding, retrieving only, or retrieving + crouching. (**A**) Although there was a tendency for infanticide by Pet1-KO virgin females, the statistical comparison showed no significant differences (Chi-square test). (**D**) A smaller number of Tph2-KO females showed crouching behavior compared to control dams. *p < 0.05 (Chi-square test). (**B,E**) Graphs showing the number of daily sessions required to retrieve the 3 pups. Infanticidal and not responding females were excluded from these quantifications. (**B**) Number of sessions required to retrieve 3 pups was similar between Pet1-KO and control virgin mice, with the majority being able to complete the task from the first session. (**E**) Tph2-KO females required more sessions than control mice to retrieve 3 pups, *p < 0.05 (mean ± SEM, Mann–Whitney test). (**C,F**) Graphs showing the number of daily sessions required by the females that performed retrieval followed by crouching over at least 2 pups continuously for more than 1 min. Both retrieving (**C**) Pet1-KO and (**F**) Tph2-KO virgin mice required more daily sessions to show crouching behavior, compared to their respective controls, **p < 0.01 (mean ± SEM, Mann–Whitney test).

These observations suggested that nursing and/or lactation defects could account for the poor survival rate of the offspring. Lactation defects can have different origins such as hormonal change or inadequate mother–pup interactions. Gestational hormones stimulate mammary gland development and milk production, while interactions with the pup and suckling provide a positive feedback that leads to further development of the mammary gland and milk ejection^35^. We thus examined the mammary gland during late gestation and after pup delivery. One day prior to delivery (PPD-1), mammary glands from gestating Pet1-KO and controls had similar weights. However, two days after pup delivery (PPD2), those of Pet1-KO mothers were less developed than controls (two-way ANOVA: Genotype x Time interaction F_1,17_ = 10.47, *p* = 0.005; Time main effect F_1,17_ = 0.7759, *p* = 0.39; Genotype main effect F_1,17_ = 9.678, *p* = 0.006; Fig. 3D). Histological analyses at PPD2 showed no difference in the structure of the mammary tissue in both genotypes, which in both cases contained milk as evidenced by the presence of lipid droplets in the acini (Fig. 3E,F), indicating that the transition from pregnancy to lactation had occurred^35^, albeit with lesser developed acini in Pet1-KO dams. Altogether, these data suggest that the deficits in reproductive performances of Pet1-KO mothers are not due to a defect in the initial development of the mammary gland during pregnancy, but could rather be a consequence of the reduced maternal crouching behavior which, in turn, would alter the positive feedback loop of suckling on milk production in parturient dams.

### Alloparental behaviors is compromised in virgin Pet1-KO and Tph2-KO females

Virgin female mice develop pup-oriented maternal behaviors, such as pup retrieval and crouching, when repeatedly exposed to neonates^15,36^. This allowed us to investigate the pup-oriented defects of hyposerotonergic females, independently of the hormonal context of pregnancy. A cohort of virgin Pet1-KO and control females (n = 14 and n = 12 respectively) was used for this experiment. Upon first exposure to pups, some mice showed infanticidal behavior toward pups (43% of Pet1-KO and of 17% in control females), but this difference was not significant (chi-square, *p* = 0.15; Fig. 4A). The other control (n = 10) and Pet1-KO (n = 8) females showed an immediate interest for the pups (Fig. 4A) and required a similar number of sessions for a complete pup retrieval behavior (MW U = 30.5, *p* = 0.31; Fig. 4B). 100% of the control females crouched onto pups, whereas 25% (2/8) of the retrieving Pet1-KO mice did not (chi-square, *p* = 0.09; Fig. 4A). Interestingly, those Pet1-KO females that crouched over pups, required more training sessions to initiate this behavior compared to controls (MW U = 7, *p* = 0.008; Fig. 4C). Similar experiments were conducted in the Tph2-KO mouse line (n = 12 Tph2-KO and n = 10 control mice). 42% of the Tph2-KO females showed infanticidal behavior compared to 10% in controls (chi-square, *p* = 0.1; Fig. 4D). One of the Tph2-KO females showed no interest for the pups and was deemed as non-responder. The remainder Tph2-KO (n = 6) and control (n = 9) females retrieved pups to the nest (Fig. 4D), but Tph2-KO mice required significantly more training sessions (MW U = 10.5, *p* = 0.02; Fig. 4E). In addition, whereas all control females showed crouching postures over the pups once in the nest, 33% (2/6) of the retrieving Tph2-KO mice never expressed this behavior (chi-square, *p* = 0.03; Fig. 4D), and those who did so, required significantly more training sessions (MW U = 1, *p* = 0.004; Fig. 4F). Thus, there are strong similarities between alloparental and maternal behaviors, with crouching behavior altered in both virgins and dams of the Pet1-KO and Tph2-KO genotypes, and pup retrieval behavior altered only in Tph2-KO virgins and dams. Altogether, these data obtained in a context free of pregnancy and parturition are consistent with the hypothesis of a common deficit in the expression of crouching/nursing behavior when 5-HT levels are constitutively reduced.

### Conditional deletion of Tph2 in the dorsal raphe nucleus (DRN) reduces pup survival

**Figure 5:**
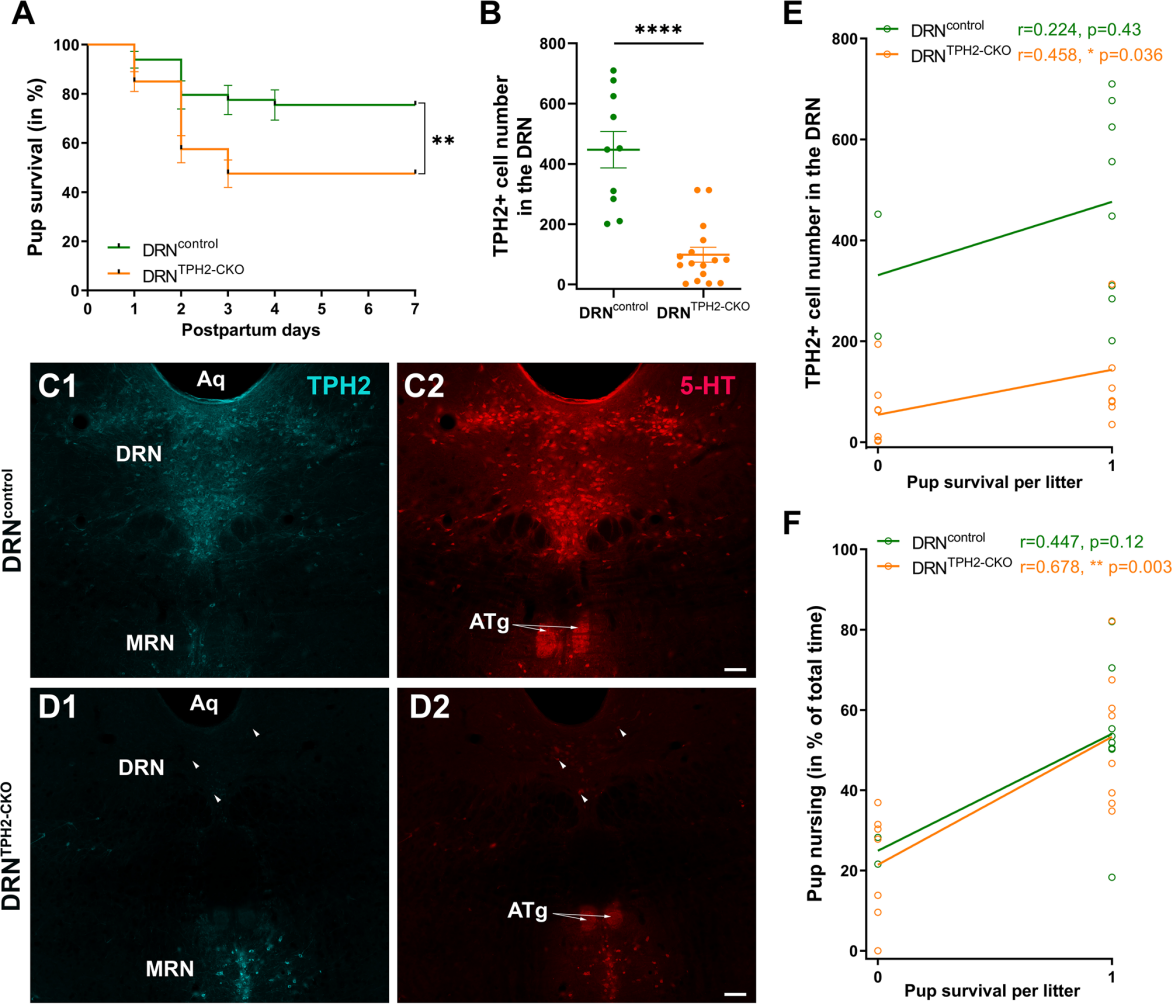
Selective 5-HT depletion from DRN neurons in experienced mothers is sufficient to reduce pup survival and nursing behavior. Primiparous Tph2^flox/flox^ and their controls (C57BL/6) were injected with AAV9.hSyn.HI.eGFPLCre.WPRE.SV40 in the dorsal raphe nucleus (DRN). Three weeks after surgery, experienced DRN^Tph2−CKO^ and DRN^control^ dams were bred again with WT males. The number of litters analyzed are n = 16 for DRN^Tph2−CKO^ dams and n = 10 for DRN^control^ dams. All litters were culled to 5 pups. (**A**) n = 8/16 DRN^Tph2−CKO^ dams lost their whole litter vs. n = 2/10 DRN^control^ dams, leading to a significant reduction of the pup survival by DRN^Tph2−CKO^ dams compared to the DRN^control^ dams; **p < 0.01 (mean ± SEM, Log-rank Mantel-Cox test). (**B**) Counting of TPH2 + neurons through 4 rostro-caudal levels encompassing the dorsal/ventral/lateral subdivisions of the DRN. A significant reduction in the number of TPH2 + neurons was found in DRN^Tph2−CKO^ dams compared to DRN^control^ dams; ****p < 0.0001 (mean ± SEM, Mann–Whitney test). (**C,D**) Representative pictures showing a broad expression of TPH2 + and 5-HT + neurons in the DRN of the DRN^control^ mothers (**C1,C2**), which is nearly absent in the DRN^Tph2−CKO^ mothers (**D1,D2**). The presence of TPH2 + (**C1–D1**) and 5-HT + (**C2–D2**) neurons in the median raphe nucleus (MRN), as well as the dense 5-HT axon innervation in the anterior tegmental nucleus (ATg, white arrows) coming from the MRN, is also observed in both cases (**C2–D2**), validating the specificity of the DRN targeting. Scale bar = 100 µm (MetaMorph v7.8, Molecular Devices, USA). (**E,F**) The scatter plots (with best-fit regression lines) show a significant correlation of pup survival per litter with the number of TPH2 + cells in the DRN (**E**) and with nursing time (**F**) in the experienced DRN^Tph2−CKO^ group. Pup survival was either fully compromised (0 on the X axis) or ≥ 80% (1 on the X axis) (Kendall’s tau test).

Because so far, most of the studies about the role of 5-HT in maternal behavior have been performed on constitutive models of 5-HT depletion, an important question was to determine whether maternal defects are the indirect consequence of altered brain development and connectivity of neural circuits involved in maternal behavior^13^ or whether they reflect a direct requirement for 5-HT neurotransmission^37^. To address this question, we conditionally ablated 5-HT production in experienced mothers by using a previously characterized Tph2^flox/flox^ mouse^20,27^. To exclude the effects of primiparity on maternal behaviors, we induced 5-HT depletion after a first maternal experience. A cohort of Tph2^flox/flox^ (n = 16) and control (n = 10) females were mated and allowed to have a first maternal experience (all pregnant females were isolated before delivery and maintained with their pups until weaning), after which they underwent surgery, with stereotaxic injections of AAV9.hSyn.HI.eGFPLCre.WPRE.SV40 in the DRN. They were subsequently placed back to the controlled mating protocol for a second parturition, which was scored as previously. A significant difference in pup survival was noted (Log-rank Mantel-Cox test: *p* = 0.003; Fig. 5A). This important lethality reflects entire offspring loss in half litters born to DRN^TPH2–CKO^ dams. No differences between DRN^control^ and DRN^TPH2−CKO^ were observed in nest building (supplementary Fig. 5A), pup retrieval (supplementary Fig. 5B), or in nursing time (supplementary Fig. 5C).

Because the extent of stereotaxic viral injections and consequent 5-HT depletion can vary across cases and thus generate phenotypic heterogeneity, we performed a histological examination of the DRN (Fig. 5C,D). In each case, serial sections were immunostained for GFP, to visualize the site of injection, and for TPH2 and 5-HT to evaluate the efficiency of recombination in different raphe subregions. In all cases, GFP positive (GFP +) cells were present in the DRN indicating successful viral delivery. Counting of TPH2-immunoreactive neurons in the DRN^TPH2−CKO^ showed a 77.8% reduction (range: from 29.7 to 99.6%) when compared to DRN^control^ mice (MW U = 8, *p* < 0.0001, Fig. 5B,C1–D1) Accordingly, a strong reduction of 5-HT immunolabeling was observed in the DRN of the DRN^TPH2−CKO^ mice (Fig. 5C2–D2) while TPH2 and 5-HT co-labeling was unchanged in the MRN indicating that recombination affected selectively the DRN (Fig. 5D1–D2). We then evaluated how the extent of 5-HT depletion and the time dedicated to nursing could affect pup survival. Since pup survival had a dichotomous distribution (0% or 80–100%), point-biserial correlations were run. Pup survival from DRN^TPH2−CKO^ dams significantly correlated with the number of TPH2 + cells in the DRN (Kendall’s tau r = 0.458, *p* = 0.036; Fig. 5E) as well as with the nursing time (Kendall’s tau r = 0.68, *p* = 0.003; Fig. 5F). Such correlations were not found in the DRN^control^ group (Fig. 5E,F)**.** In addition, the number of TPH2 + cells in the DRN and the nursing time were also correlated (spearman r = 0.4, *p* = 0.05; supplementary Fig. 5D). These data indicate that despite prior successful prior reproductive experience, acute reduction of 5-HT neurotransmission from DRN alters the adequate initiation of maternal behavior, and more specifically the expression of crouching/nursing behavior.

## Discussion

Present data show that adult depletion of 5-HT transmission from the DRN in experienced mothers is sufficient to decrease pup survival. In addition, the comparison of maternal behavior in three different genetic models of 5-HT depletion allows to pinpoint that nursing defect are most likely responsible for this enhanced pup lethality. Finally, comparison of parturient and alloparental behaviors suggests that this defect is the consequence of a reduced expression of the crouching behavior over pups rather than a gestational or parturitional hormone dysregulation.

Behavioral phenotypes, such as maternal care are not only species-specific, but strongly depend on the genetic background within a given species^15,38,39^. So far, all the studies on the role of 5-HT on maternal behavior have been performed on genetic models of 5-HT depletion generated on a mixed 129 or FVB/N and C57BL/6 genetic background^11,12,14^. The backcross of the Pet1-KO and Tph2-KO mouse lines to a pure C57BL/6 background^19,26^ allowed us to compare maternal behavior independently of this variable. Our observations were consistent with previous studies, showing that litter survival is highly compromised in the Pet1-KO and Tph2-KO mouse lines. However, our detailed analysis draws a somewhat different and more nuanced picture of their maternal behavior. To our surprise, many aspects of maternal responsiveness to pups in Pet1-KO dams with pure C57BL/6 genetic background were not perturbed: pup retrieval, nest building, huddling of pups into the nest were similar to controls, unlike what had previously been observed in the Pet1-KO mice with mixed background^12^. Interestingly, differences in anxiety-like behaviors have also been reported between these strains^38^, with the Pet1-KO on a C57BL/6 genetic background showing less anxiety than the Pet1-KO on a mixed 129 and C57BL/6 background, likely contributing to the observed differences in maternal behavior. In the Tph2-KO with a C57BL/6 genetic background, the analyses of maternal behavior showed a larger spectrum of alterations such as imperfect nest building, defects in pup huddling and retrieval, that were similar to previous descriptions on a mixed background^11^, albeit less pronounced.

Comparisons between constitutive Pet1-KO and Tph2-KO indicate that pup retrieval and huddling are more affected in complete rather than in partial 5-HT depleted mouse models, suggesting a correlation between the level of 5-HT depletion and the degree of maternal neglect. This link was further strengthened by our evaluation of alloparental behavior of virgin Pet1-KO and Tph2-KO females, the latter having a stronger phenotype. Indeed, *Tph2* inactivation leads to a drop of 99% of the brain 5-HT levels^11,16,20,40^, whereas *pet1* inactivation causes a 75% depletion of brain 5-HT^18^, with some hot spots of residual 5-HT axon innervation in the amygdala and hypothalamus^19^. Differences in the mechanism and specificity of 5-HT depletion may also contribute to the different maternal phenotypes in the two models. In the Tph2-KO mice, 5-HT neurons in the raphe nuclei develop normally and express all the characteristic molecular markers (e.g. SERT, VMAT2, VGLUT3, etc.), whereas in the Pet1-KO mice, raphe progenitors are maintained in an arrested state of differentiation, and the remaining Pet1-resistant 5-HT neurons show reduced expression of the genes necessary for 5-HT homeostasis and for glutamate or neuropeptide synthesis^17,41^.

Because 5-HT production in the brain is reduced during the entire development of Pet1-KO and Tph2-KO mice, one could hypothesize that altered parental behaviors in female mice is the consequence of an altered development of specific neural circuits controlling pup-oriented behaviors^2,3,15^. Indeed, although the general organization and structure of the developing brain may not require 5-HT, complete or partial 5-HT depletion can have subtle effects on the fine wiring and homeostatic functioning of neural circuits, leading to abnormal adult behaviors^6,38,42,43^. However, our study of maternal behavior after conditional 5-HT depletion in adult dams rule out this possibility. Moreover, the fact that 5-HT depletion was done after a normal maternal experience indicates that this neurotransmitter is not affecting learning/plasticity mechanisms involved in parental behaviors, but that normal 5-HT transmission is actually required at the time of gestation and parturition for adequate maternal care and pup survival. The 5-HT raphe nuclei innervate all the brain targets that are involved in maternal behaviors (MPOA, BNST, hypothalamus, etc.)^8^. We found that selective 5-HT depletion in the DRN was sufficient to reproduce the phenotype of the Pet1-KO mothers with reduced survival of litters and nursing deficits that were correlated to the extent of the reduction in TPH2-immunopositive neurons in the DRN. In our experiments, the 5-HT depletion was highly restricted to the entire DRN (i.e. the dorsal, ventral and lateral subdivisions), whereas the MNR was not affected. This indicates that axon projections from the DRN are essential for initiating an adequate nursing behavior, although the specific targets remain to be identified. Conversely, induction of DRN-specific Tph2 deletion had no effect on pup retrieval behavior or pup huddling, indicating that 5-HT neurons from DRN are not required for the expression of these maternal motivated behaviors. It will be interesting to target selectively the MRN to determine whether these other components of the maternal deficiencies observed in the Tph2-KO can be reproduced. In this regard, recent genetic strategies to target subsets of the DRN could be of great interest^24,44–46^.

Our observations indicate that one component of the maternal repertoire, the nursing behavior is consistently affected in all the models analyzed. Nursing is the behavioral concomitant of lactation, and consists of a sequence of mother–pup interactions, that have been well described in the rat^1^. Nursing is elicited by proximal suckling and ventral somatic sensory stimulation from pups^47,48^. Suckling in turn triggers prolactin (PRL) and oxytocin (OT) release to stimulate milk production and ejection respectively, and promote dam’s quiescence and adoption of the kyphotic nursing posture. Nursing implies crouching over the pups, a behavior which is activated by physical interactions involving snout tactile sensations of the mother by her offspring. Thus, touch plays an essential role, while in comparison, olfactory, auditory or visual cues coming from pups are not necessary, but are important to maintain the maternal dam’s interest for its progeny^15,47^. Mechanisms mediating crouching and nursing behavior have been less studied in mice, and although maternal behaviors show qualitative similarities in mice and rats, there are differences between both species at least in the relative importance of sensory inputs, to elicit maternal care^15^. In our study, we found that both primiparous Pet1-KO and Tph2-KO dams showed a reduction in the time dedicated to crouching/nursing, associated with lack of the high crouching posture (kyphosis), characterized by rigid limb support and ventroflexion^15,49^. Mutant dams were found either lying close to their pups, or with a low crouching posture without the ventroflexion, or with a passive nursing posture without limb support. In contrast, the percentage of time dedicated to pup licking was unchanged in the different hyposerotonergic models indicating that there is not a global defect in maternal care. Interestingly, although maternal behaviors, once acquired, become more efficient with subsequent reproductive experience^34^, secondiparous Pet1-KO dams showed no improvement of the time spent crouched over the pups, suggesting a specific role of 5-HT for the expression of crouching/nursing behavior. The specific nursing defect we observed could suggest a defective lactation in the 5-HT depleted mothers because 5-HT has a stimulatory effect on PRL^50^ and probably on OT^51,52^ release, through projections to hypothalamic nuclei. However, the crouching/nursing phenotype that we noted here differs from what has been described in PRL-KO and OT-KO mice. Indeed, PRL-deficiency prevents the normal development of mammary glands during gestation^53^ and OT-KO mothers encourage suckling by progeny, although no milk can be found in the pup’s stomachs due to milk ejection failure^54^. In contrast, mammary glands of pregnant Pet1-KO mice grew normally and contained milk, but they became hypotrophic 48 hs postpartum. Moreover, we could confirm the presence of a milk pouch in pups from Pet1-KO and Tph2-KO litters, as noted in previous reports^11,12^, although its size was reduced few hours after delivery, and further decreased until death. Interestingly, nursing and lactation can be independently regulated. As already mentioned, crouching response occurs in rodent dams that are not capable of milk production or ejection^47,54,55^. Secondly, pup exposure stimulates the expression of crouching behavior in virgin mice and rat females, which can adopt the kyphosis posture and stay quiescent to favor pup attachment to their nipples^15^. We found that control females from the Pet1 and Tph2 mouse lines showed a nursing-like posture as early as the first training session, whereas both Pet1-KO and Tph2-KO virgin mice required a longer exposure to pups before initiating a crouching behavior. In contrast, virgin Prl-KO and OT-KO females both show normal crouching behavior^53,56^. Altogether, these data indicate that 5-HT neuromodulation is requested for the proper expression of crouching behavior by lactating mothers and by virgins. The precise brain regions that are involved in this effect remain to be identified. Some lesion studies indicate that structures such as the periaqueductal gray, which receives a 5-HT innervation from the different subdivisions of the DRN^8^, could be one of them, since it has been shown to control kyphotic posture^47,57^. It will be of interest in future studies to identify specific 5-HT receptors and neural circuits involved in this behavior with chemogenetic or optogenetic approaches. On a translational point of view, our findings may be related to observations of reduced maternal/infant interactions in depression, the understanding of which could lead to adapted behavioral therapies.

## Supplementary Information

Supplementary Figure 1.

Supplementary Figure 2.

Supplementary Figure 3.

Supplementary Figure 4.

Supplementary Figure 5.

Supplementary Legends.

## Data Availability

The datasets generated during and/or analyzed during the current study are available from the corresponding author on reasonable request.
