# Supplementary figures and images for: Dorsal raphe serotonin neurotransmission is required for the expression of nursing behavior and for pup survival
Source: Sci Rep. 2021 Mar 16;11:6004. doi: 10.1038/s41598-021-84368-6 (PMC7966367; doi:10.1038/s41598-021-84368-6)

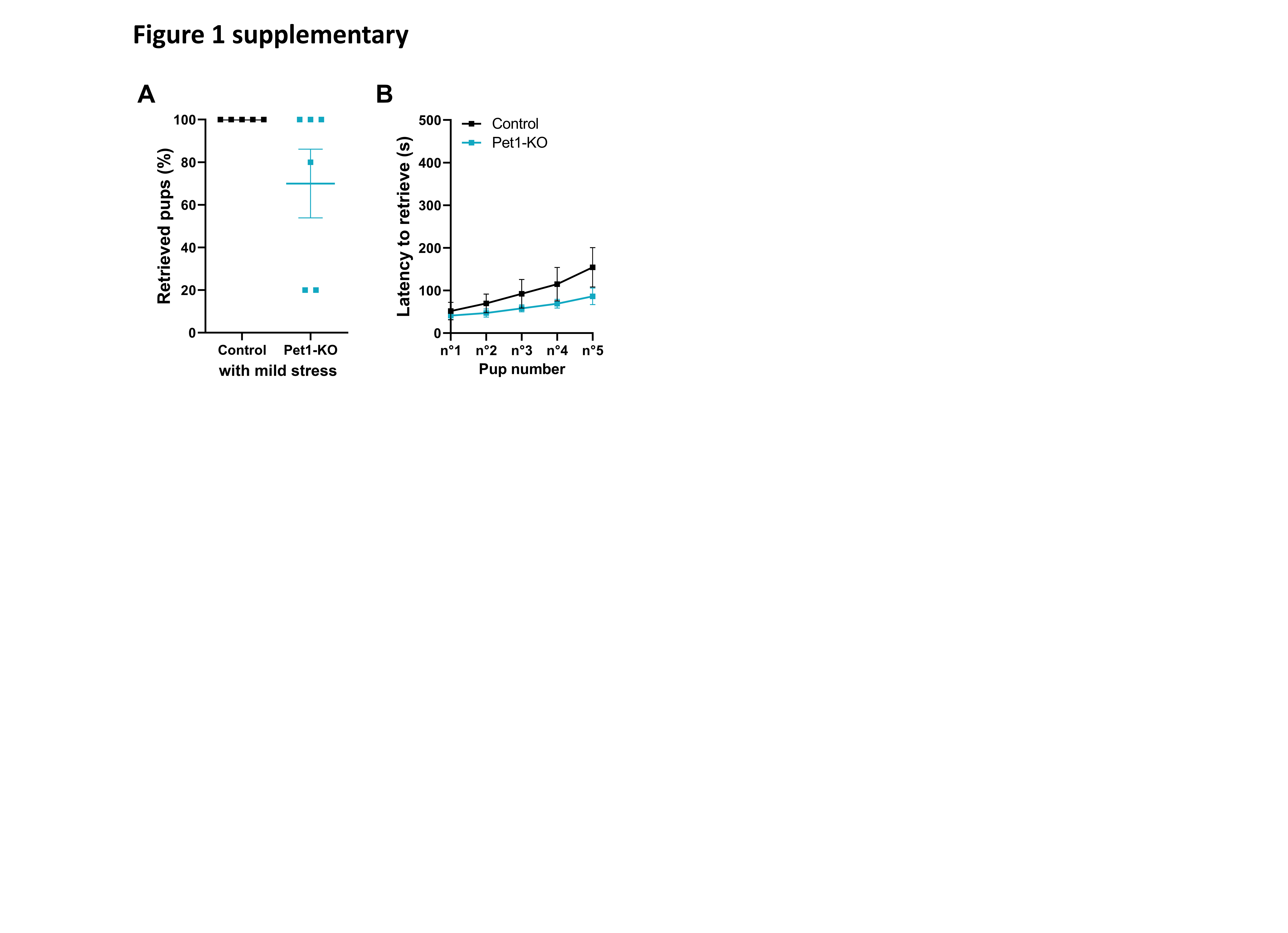

Supplement: Supplementary file 1 — Supplementary Figure 1. [file 41598_2021_84368_MOESM1_ESM.jpg]

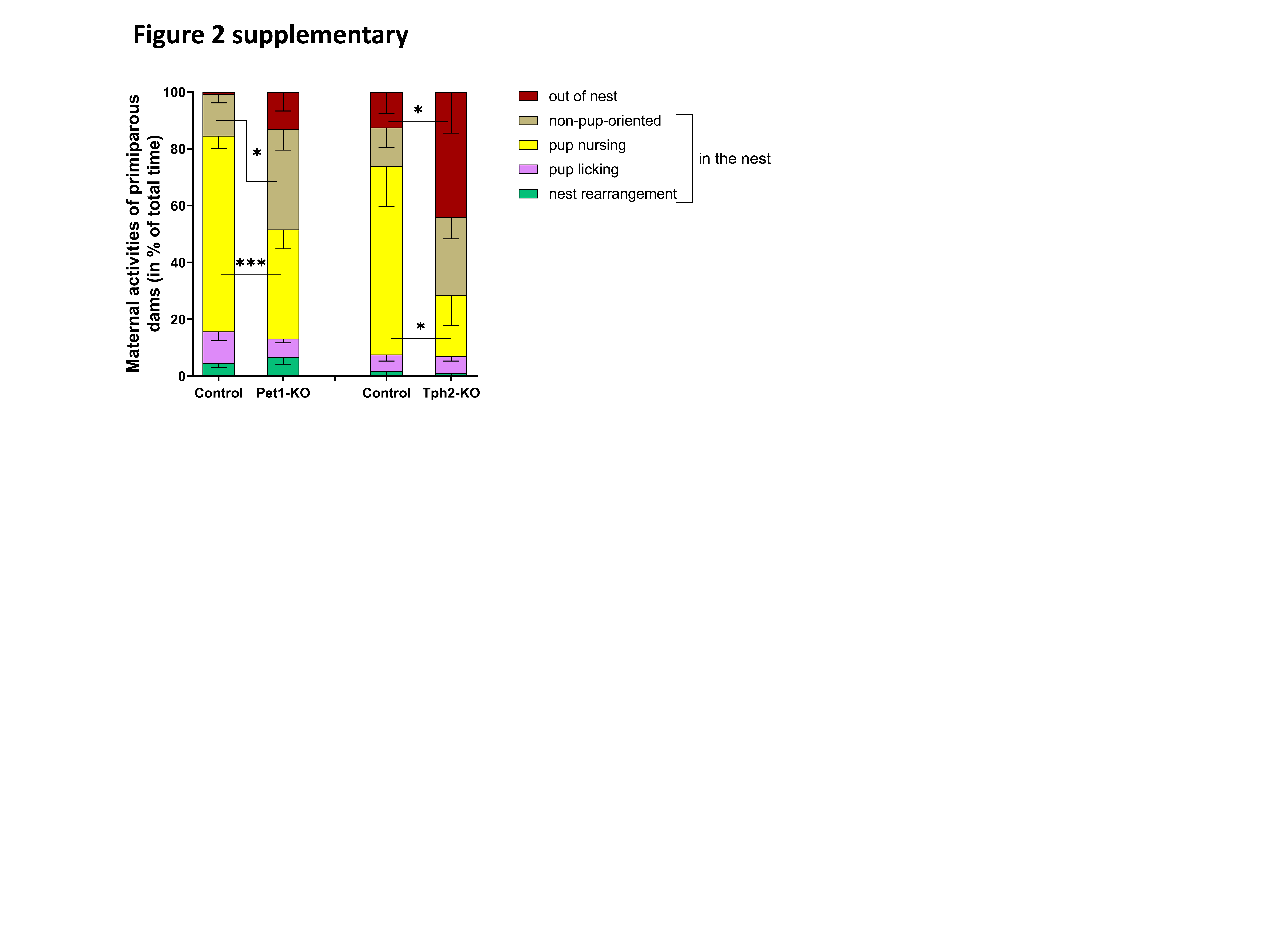

Supplement: Supplementary file 2 — Supplementary Figure 2. [file 41598_2021_84368_MOESM2_ESM.jpg]

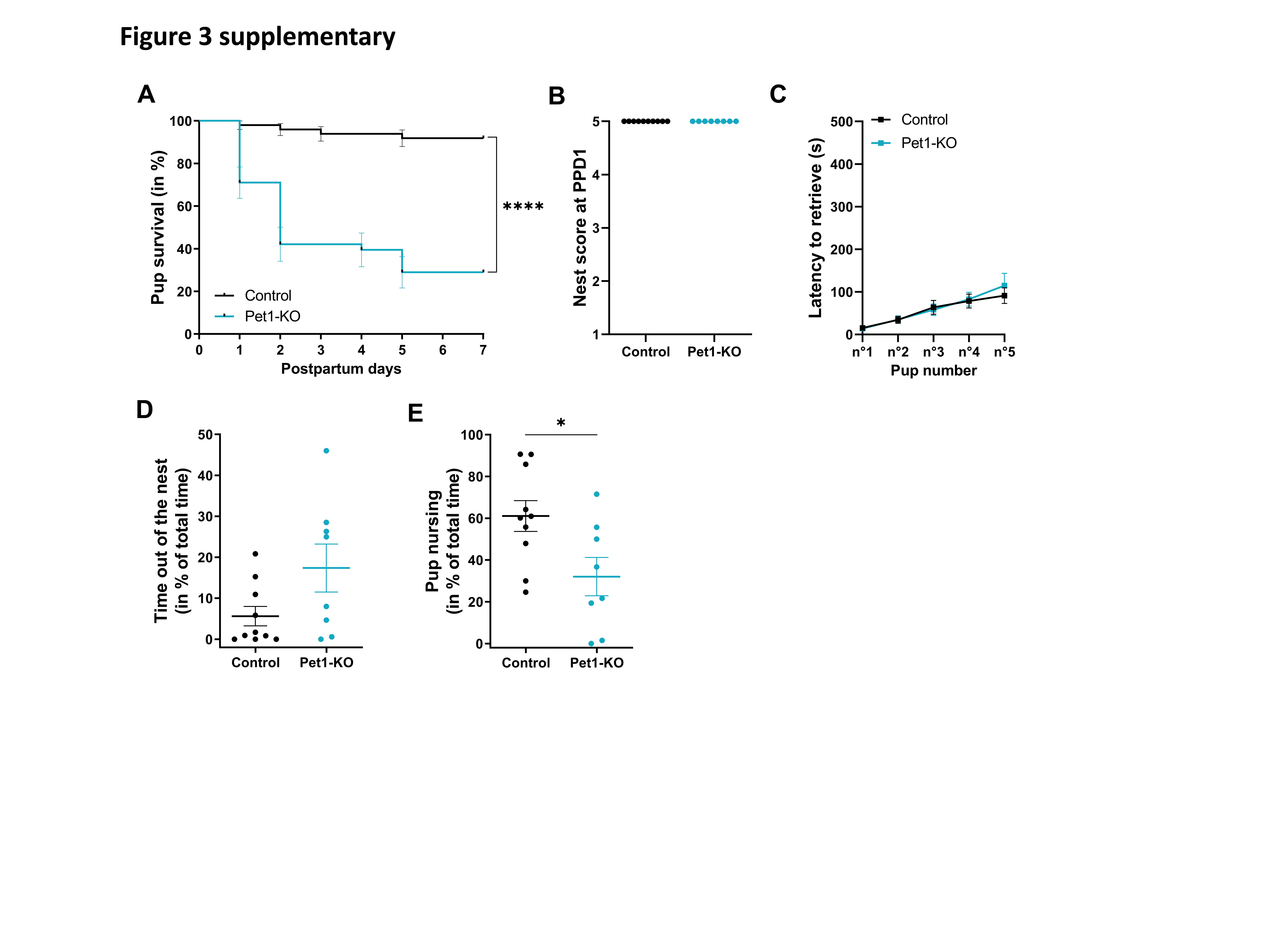

Supplement: Supplementary file 3 — Supplementary Figure 3. [file 41598_2021_84368_MOESM3_ESM.jpg]

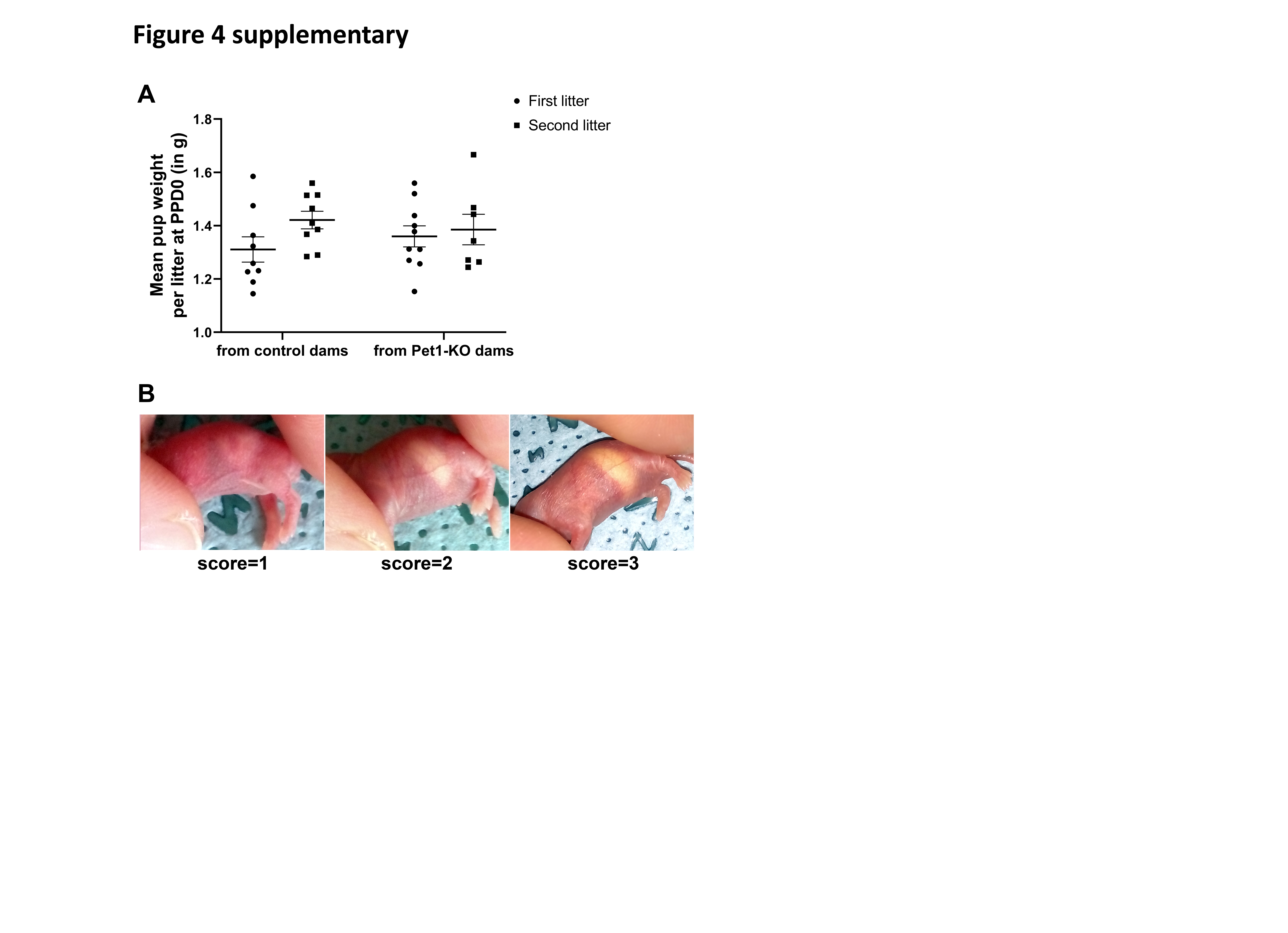

Supplement: Supplementary file 4 — Supplementary Figure 4. [file 41598_2021_84368_MOESM4_ESM.jpg]

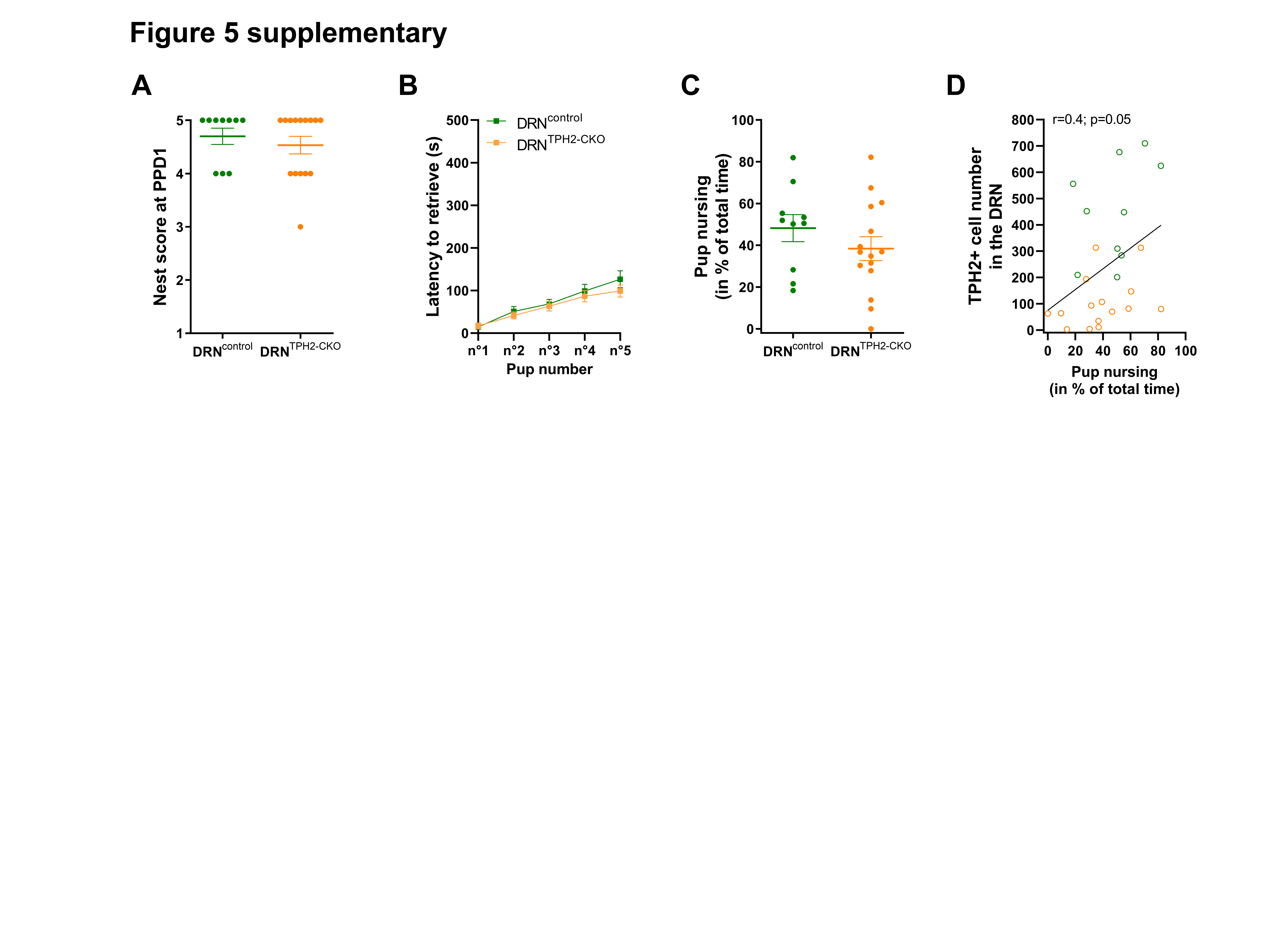

Supplement: Supplementary file 5 — Supplementary Figure 5. [file 41598_2021_84368_MOESM5_ESM.jpg]
