# Supplementary material for: Dorsal raphe serotonin neurotransmission is required for the expression of nursing behavior and for pup survival
Source: Sci Rep. 2021 Mar 16;11:6004. doi: 10.1038/s41598-021-84368-6 (PMC7966367; doi:10.1038/s41598-021-84368-6)
Supplement: Supplementary file 6 — Supplementary Legends. [file 41598_2021_84368_MOESM6_ESM.pdf]

Dorsal raphe serotonin neurotransmission is required for the expression of nursing behavior and for pup survival

Aude Muzerelle[1], Mariano Soiza-Reilly[1,2], Cornelia Hainer[3], Pierre-Louis Ruet[1], Klaus-Peter Lesch[4,5,6], Michael Bader[3,7,8,9], Natalia Alenina[3,7,10,11,*], Sophie Scotto-Lomassese[1,13,*] and Patricia Gaspar[1,12, 13,*]

Supplementary Figure 1. Normal maternal responsiveness in primiparous Pet1-KO mothers after mild stress

(**A**) The number of retrieved pups (in %) was not different between Pet1-KO and control dams, despite a prior exposure to a mild stress (mean ± SEM; MW U=7.5, p=0.18). (**B**) The latency to retrieve (in seconds) was not different between Pet1-KO and control dams, despite a prior exposure to a mild stress (mean ± SEM; repeated-measures two-way ANOVA: genotype x pup number interaction $F_{4,26}$=1.438, p=0.25; Genotype main effect $F_{1,9}$=0.8936, p=0.37).

Supplementary Figure 2. Comparison of the maternal activities at PPD0 from primiparous Pet1-KO and Tph2-KO mothers

Histograms show the time dedicated to pup-oriented and non-pup-oriented activities of the primiparous Pet1-KO and Tph2-KO mothers compared to their respective controls (in % of total time). Presence in the nest was significantly reduced for the Tph2-KO mothers but not for the Pet1-KO mothers. When all the mothers were in the nest with all or only a fraction of their pups, no differences were found in pup licking and nest rearrangement activities between mutant and control mothers of the two mouse lines. By contrast, both Pet1-KO and Tph2-KO mothers spent significantly less time to pup nursing compared to controls. In addition, Pet1-KO mothers spent significantly more time to non-pup-oriented activities inside the nest, such as sleeping or self-grooming (MW U=17, p=0.04); * p<0.05, ** p<0.01 (mean ± SEM, Mann-Whitney test).

Supplementary Figure 3. Maternal experience does not improve reproductive success and nursing behavior in Pet1-KO females

Primiparous Pet1-KO and control (Pet1$^{+/-}$) were mated to WT males. The number of litters analyzed was n=8 for Pet1-KO dams and n=10 for control dams. All litters were culled to 5 pups. (**A**) Survival of pups raised by Pet1-KO dams was strongly reduced; **** p<0.0001 (mean ± SEM, Log-rank Mantel-Cox test). (**B**) Nest building score at PPD1 was similar between both groups of dams (mean ± SEM, MW U=40, p>0.99). (**C**) No difference in the latency to retrieve the 5 pups into the nest was found between secondiparous Pet1-KO and control mothers (mean ± SEM, two-way ANOVA: genotype x pup number interaction $F_{4,54}$=0.8489, p=0.50; Genotype main effect $F_{1,15}$=0.1101, p=0.74). (**D-E**) Time spent outside the nest did not differ between both groups of dams, but the Pet1-KO dams dedicated less time to nurse their pups; * p<0.05 (mean ± SEM, Mann-Whitney test).

Supplementary Figure 4. Weight at birth and semi-quantitative scoring of the milk pouch

(**A**) There are no differences in the weight of pups per litter at birth between Pet1-KO and control dams, irrespective of maternal experience (mean per litter ± SEM; two-way ANOVA: Genotype x Maternal Experience interaction $F_{1,11}$=1.381, p=0.27; Genotype main effect $F_{1,20}$=0.01917, p=0.89; Maternal Experience main effect $F_{1,11}$=2.783, p=0.12). (**B**) Representative pictures of the semi-

quantitative milk pouch scoring from PPD1 pups. Left panel: score 1= pouch with little milk visible. Middle panel: score 2= visible milk pouch. Right panel: score 3= pouch full of milk. Image source: A. Muzerelle and S. Scotto-Lomassese (UMR-S 1270).

Supplementary Figure 5. Maternal responsiveness of experienced $DRN^{control}$ and $DRN^{TPH2\text{-}CKO}$ mothers

(**A**) Nest building score at PPD1 was similar between $DRN^{Tph2\text{-}CKO}$ and $DRN^{control}$ dams (mean ± SEM, Mann-Whitney test U=62.5, p=0.56). (**B**) No difference in the latency to retrieve 5 pups into the nest was found between both groups (mean ± SEM, repeated-measures two-way ANOVA: genotype x pup number interaction $F_{4,87}$=1.498, p=0.21; Genotype main effect $F_{1,22}$=0.5189, p=0.48). (**C**) No difference in the percent of time dedicated to pup nursing during 20 min observation was found between both groups (mean ± SEM, Mann-Whitney test U=57, p=0.34). (**D**) The scatter plot (with best-fit regression line) shows a significant correlation between the number of TPH2+ cells in the DRN and the nursing time of experienced $DRN^{Tph2\text{-}CKO}$ (orange circle) and $DRN^{control}$ (green circle) dams (Spearman test).
